# Exosomes derived from heat shock preconditioned bone marrow mesenchymal stem cells alleviate cisplatin-induced ototoxicity in mice

**DOI:** 10.1186/s13036-022-00304-w

**Published:** 2022-09-29

**Authors:** Tao Yang, Wei Li, Anquan Peng, Qin Wang

**Affiliations:** Department of Otolaryngology and Head &Neck Surgery, The Second Xiangya Hospital, Central South University, Changsha, 410011 Hunan China

**Keywords:** Bone marrow mesenchymal stem cells, Exosomes, Heat shock precondition, Cisplatin-induced ototoxicity

## Abstract

NOD-like receptor family pyrin domain containing 3 (NLRP3) inflammasome contributes to the development of cisplatin-induced ototoxicity. Whether heat shock pretreatment could be utilized to up-regulate 70 kilodalton heat shock proteins (HSP70) expression in bone marrow mesenchymal stem cells (BMSCs)-derived exosomes (HS-BMSC-Exo) to alleviate cisplatin-induced ototoxicity is deciphered in this study. Heat shock pretreatment was performed on BMSCs to induce HS-BMSC-Exo, which were further trans-tympanically administrated into cisplatin intraperitoneally injected C57BL/6 mice. Auditory brainstem response (ABR) was assessed to indicate auditory sensitivity at 8, 16, 24, and 32 kHz. Myosin 7a staining was utilized to detect the mature hair cells. The relative expressions of the NLRP3 inflammasome complex were determined with Western blot in the cochlea. Diminished auditory sensitivity and increased hair cell loss could be observed in the cisplatin exposed mice with increased content of interleukin (IL)-1β, IL-6, tumor necrosis factor (TNF)-α, NLRP3, ASC, cleaved caspase-1, and pro-caspase-1, and decreased content of IL-10, which could be reversed by HS-BMSC-Exo or BMSC-Exo administration. It was worth noting that HS-BMSC-Exo demonstrated more treatment benefits than BMSC-Exo in cisplatin-induced ototoxicity. Heat shock precondition may provide a new therapeutic option to produce exosomal HSP70, and HS-BMSC-Exo could be utilized to relieve cisplatin-induced ototoxicity.

## Introduction

As a highly effective chemotherapy drug, cisplatin is generally utilized in solid malignant tumors. However, the severe side effects of ototoxicity, nephrotoxicity, and bone marrow depression limit the clinical application (Kros and Steyger, 2019; Perše, 2021). The incidence of cisplatin-induced ototoxicity can range from 20 to 70%, dependent on different reports [15, 17]. Inflammation, oxidative stress, cellular uptake of cisplatin, autophagy, and necroptosis could be attributed to progressive hair cell death and consequent hearing loss [4]. Thus, to further deciphering the potential mechanism of inflammation-induced cisplatin ototoxicity is vital in clinical practice.

NOD-leucine-rich repeat and pyrin-containing protein 3 (NLRP3) inflammasome is a vital player in inflammation or innate immunity, comprising NLRP3, apoptosis-associated speck-like protein (ASC), and pro-caspase-1, which sensors microbes or damage-associated molecular patterns to regulate the activation of caspase-1-dependent release of interleukin (IL)-1β and IL-18 [10, 19]. Heat shock protein 70 (HSP70) can directly interact with NLRP3 to prohibit the activation of NLRP3 inflammasome [1]. On the other hand, NLRP3 inflammasome inhibition with MCC950 could relieve cisplatin-induced renal fibrosis by diminished oxidative stress and inflammation [8]. Whether NLRP3 inflammasome activation leads to the development of cisplatin-induced ototoxicity is deciphered in this study.

Heat shock precondition could promote the survival of transfused mesenchymal stem cells in liver ischemia/reperfusion injury or cisplatin-induced granulosa cell apoptosis model [13, 18]. Exosomes derived from heat-shocked utricles could carry HSP70 to improve the survival of hair cells in aminoglycoside antibiotic neomycin-induced ototoxicity [2]. All of these researches indicate the possibility of treating NLRP3 inflammasome-induced cisplatin ototoxicity via HSP70 induction and/or administration.

In this study, bone marrow mesenchymal stem cells (BMSCs) are preconditioned with heat shock to induce the enrichment of HSP70 in the secreted exosomes, which are further transfused to alleviate cisplatin-induced ototoxicity in mice by modulating the NLRP3 inflammasome in the cochlea. Our framework methodology to construct heat shock-induced BMSCs derived exosome is a valuable tool to deliver HSP70. Targeted inhibition of NLRP3 inflammasome with exosomal HSP70 may perturb the progress of cisplatin-induced ototoxicity.

## Methods & materials

### BMSCs isolation

BMSCs were isolated as described in the previous report [22]. In brief, bone marrow cells were flushed out from the bone marrow of C57BL/6 mice with 2% heat-inactivated fetal bovine serum (FBS, Gibco) and centrifugated in density gradient to isolate bone marrow mononuclear cells. Then, CD11b^+^ microbeads (StemCell Technologies) were utilized to select mononuclear granulocytes positively, and the remaining cells were further cultured for 72–96 h. The attached BMSCs were used for subsequent experiments.

### FACS analyzing BMSCs

BMSCs were incubated with FITC conjugated CD44, PE-conjugated CD105, PerCP-Cy 5.5-conjugated CD34, and PE-Cy7-conjugated CD45 (10 μg/ml: BD Biosciences) for 20 min at room temperature, which were further fixed with 2% formaldehyde/ phosphate-buffered saline (PBS) solution on ice. Isotype controls were also utilized to confirm the specific staining. At least 50,000 singlet events were detected on a BD Facscanto II Flow Cytometer.

### Heat shock-preconditioned BMSCs

The procedure for preparing heat shock-preconditioned BMSCs was performed as previously described [9]. In brief, BMSCs in the 3rd passage were exposed to heat shock conditions (42 °C water bath) for one hour in exosome-depleted FBS (Gibco) and subsequently normally cultured (37 °C, 5% CO_2_). The control group was normally cultivated without heat shock pretreatment.

### Cisplatin-induced ototoxicity in mice

C57BL/6 mice (eight-week-old) were purchased from Vital River Laboratory Animal Ltd. (Beijing, China). In brief, cisplatin (30 mg/kg, 1 mg/ml) was intraperitoneally injected to construct cisplatin-induced ototoxicity as described (Tsai et al., 2021). All the study protocols were approved by the the Second Xiangya Hospital, Central South University.

### Exosomes isolation and testification

BMSCs derived exosomes using the previously described protocol [2, 20]. In brief, a low-speed centrifuge (300 × g, 10 min, 4 °C) was utilized to remove cells, and a high-speed centrifuge (10,000 × g, 30 min, 4 °C) was utilized to isolate large vesicles and additional cellular debris. Ultracentrifuge (100,000 × g, 70 min, 4 °C) was further applied to sediment exosomes on Optima MAX-XP ultracentrifuge (Beckman Coulter), which were resuspended in PBS or culture medium by trituration.

The distribution of exosomes size was revealed by nanoparticle tracking analysis with NanoSight NS300 equipment (Malvern Instruments LTD). The single membrane structure was revealed with transmission electron microscopy (TEM) analysis with a JEM-1400Flash Electron Microscope (Jeol). The surface markers of the exosomes (CD63, CD9, and Alix) were detected with Western blots.

### Auditory brainstem response

Auditory brainstem response (ABR) was detected with the TDT System III apparatus (Tucker Davies Technologies) to measure the hearing threshold seven days post cisplatin administration. In brief, acoustic stimuli (8, 16, 24, and 32 kHz; 100 ms duration; intensities ranged from 10 to 100 dB) were directly introduced into the ear canal of cisplatin-induced ototoxic mice. The detecting electrodes were separately placed on the vertex, below the right ear, and below the pinna of the left ear. Subdermal needle electrodes were utilized to record the ABR data. After ABR measurement, cochlear tissues were collected for expression detection and immunofluorescence staining analyses.

### Reverse-transcription PCR (RT-PCR)

Total RNA was extracted from BMSCs with Trizol (Invitrogen), and one microgram RNA was reverse transcribed with Applied Biosystems High-Capacity cDNA Reverse Transcription kits. The reverse-transcription PCR (RT-PCR) reaction was constructed in a 15-µl volume (0.375 μM primer, 0.5 μl cDNA) with 2 × FastStart Universal SYBR Green Master Mix (Roche Ltd.). An initial denaturation of 95 °C for 10 min, followed by 40 cycles of 95 °C for 15 s, and 60 °C for 1 min to detect the amplification on an ABI STEPONE System (Applied Biosystems). The relative expression was quantified with the comparative ΔCT method, and β-actin was utilized as internal genomic control. The primers utilized: HSP70, forward, 5’-CAACGTGCTCATCTTCGACC-3’, reverse, 5’-GGCTGATGTCCTTCTTGTGC-3’; β-actin, forward, 5’-CACGATGGAGGGGCCGGACTCATC-3’, reverse, 5’-TAAAGACCTCTATGCCAACACAGT-3’.

### Western blotting

The lysates derived from BMSCs, BMSC-Exo, HS-BMSC-Exo, and the middle turns of the cochlear tissues were separated by 10% SDS-PAGE and transferred to PVDF membranes, which were further blocked with 5% non-fat milk and incubated with primary antibodies against HSP70, CD63, CD9, Alix, pro-caspase-1, cleaved caspase-1, NLRP3, ASC, and GAPDH (Santa Cruz). Peroxidase-conjugated secondary antibody (Sigma-Aldrich, 1:2000 dilution) was incubated for 2 h at room temperature, and a Pierce™ Enhanced Chemiluminescence (Thermo Scientific) was applied to obtain the chemiluminescence signal. GAPDH was utilized as an internal control to normalize the relative expression of interest genes with NIH-Image J1.51p 22.

### Myosin staining

Cochlea tissues were fixed with 4% paraformaldehyde and permeabilized with 1% Triton X-100 for two hours at room temperature. The permeabilized tissues were further blocked with 5% goat serum, incubated with anti-myosin 7a antibody (Santa Cruz), and subsequently incubated with FITC-conjugated secondary antibody (Abcam). Images were observed under a Nikon 80*i* microscope.

### Enzyme-linked immunosorbent assay (ELISA)

The content of interleukin (IL)-1β, tumor necrosis factor α (TNF-α), and IL-6 in the cochlea was measured with corresponding ELISA kits (eBioscience) according to the manufacturer’s instructions. All the samples and standards were measured with a SpectraMax M5 microplate reader (Molecular Devices) at the wavelength of 450 nm.

### Statistical analysis

Quantitative parameters between different groups were assessed with one-way ANOVA followed Tukey’s multiple comparisons test or Mann Whitney test. The significance level was set as *p*-value < 0.05. All statistical analyses were performed with GraphPad Prism.

## Results

### Heat shock precondition induces up-regulated HSP70 expression in BMSCs

**Fig. 1:**
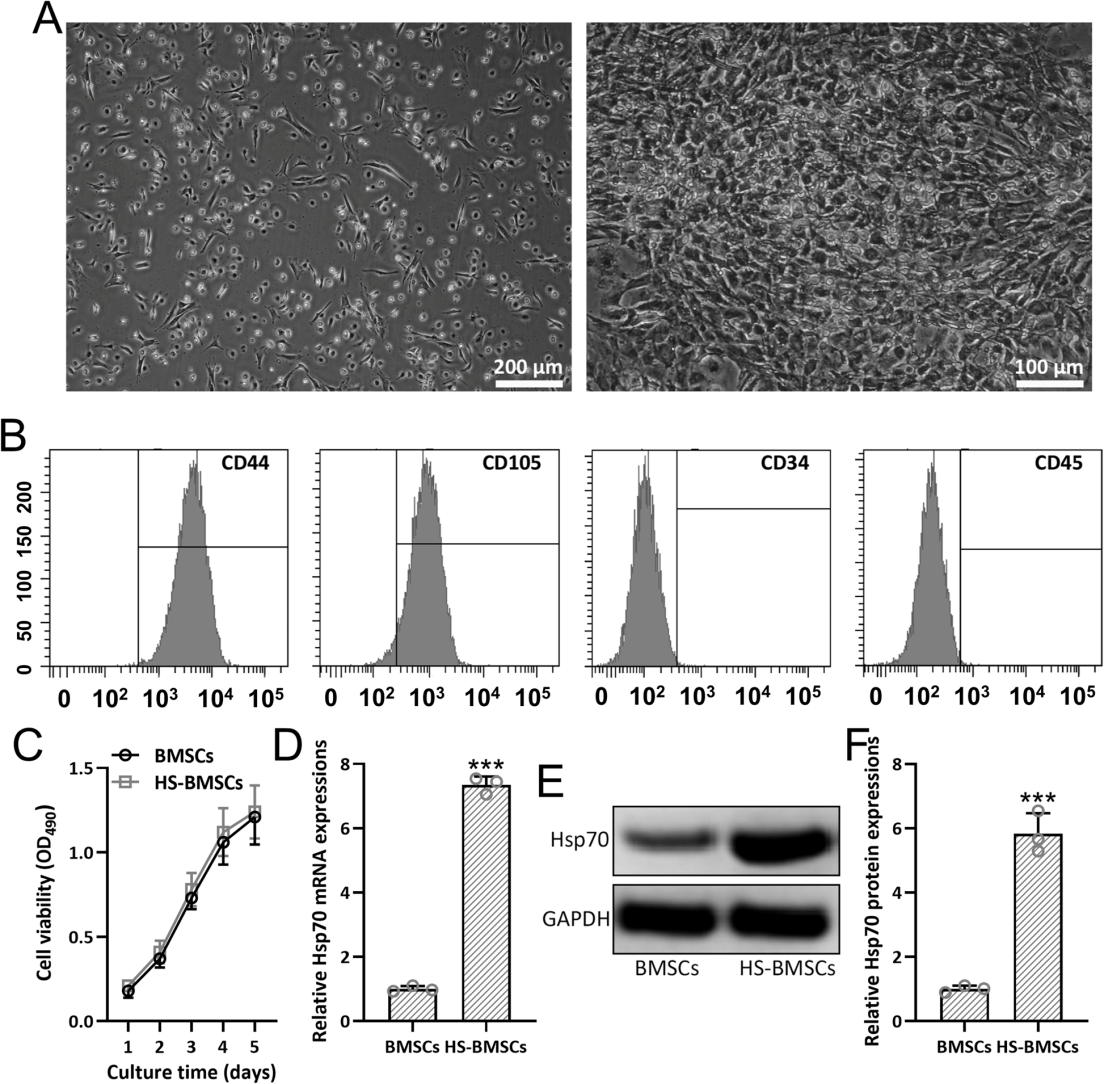
Identification of primary BMSCs from mice and the expressions of HSP70 in BMSCs after heat shock treatment. **A** Light microscopy images of bone marrow mesenchymal stem cells (BMSCs) cultured for 3 days (left) and 7 days (right). **B** Positive expressions of CD44, CD105, and negative expressions of CD34, CD45 were detected with flow cytometry. **C** CCK-8 was used to measure the cell viability of BMSCs with or without heat shock precondition. **D** The relative expression of HSP70 was assayed in heat shock preconditioned BMSCs with RT-PCR. Western blotting was used to measure the expressions of HSP70 from heat shock preconditioned BMSCs (**E**). The data were shown with mean ± SD. *n* = 3 for each group. ***p < 0.001 compared to control. Mann Whitney test

Heat shock preconditioned BMSCs were cultured for the indicated time, which demonstrated spindle-shaped morphology as expected (Fig. 1A). Positive expressions of CD44, CD105, and negative expressions of CD34, CD45 confirmed the mesenchymal stem cell lineage characteristics of BMSCs (Fig. 1B). When compared with normal cultured BMSCs, heat shock preconditioned BMSCs showed no significant difference in cell viability (Fig. 1C). As expected, the relatively increased mRNA expressions of HSP70 was detected in heat shock preconditioned BMSCs compared with normal cultured BMSCs (Fig. 1D). Western blot analysis also testified the up-regulated HSP70 level in the heat shock preconditioned BMSCs (Fig. 1E and F). All of these results indicated that heat shock precondition did not alter the viability of BMSCs, but induce up-regulated HSP70 expression.

### Heat shock precondition induces enriched HSP70 content in BMSCs derived exosomes

**Fig. 2:**
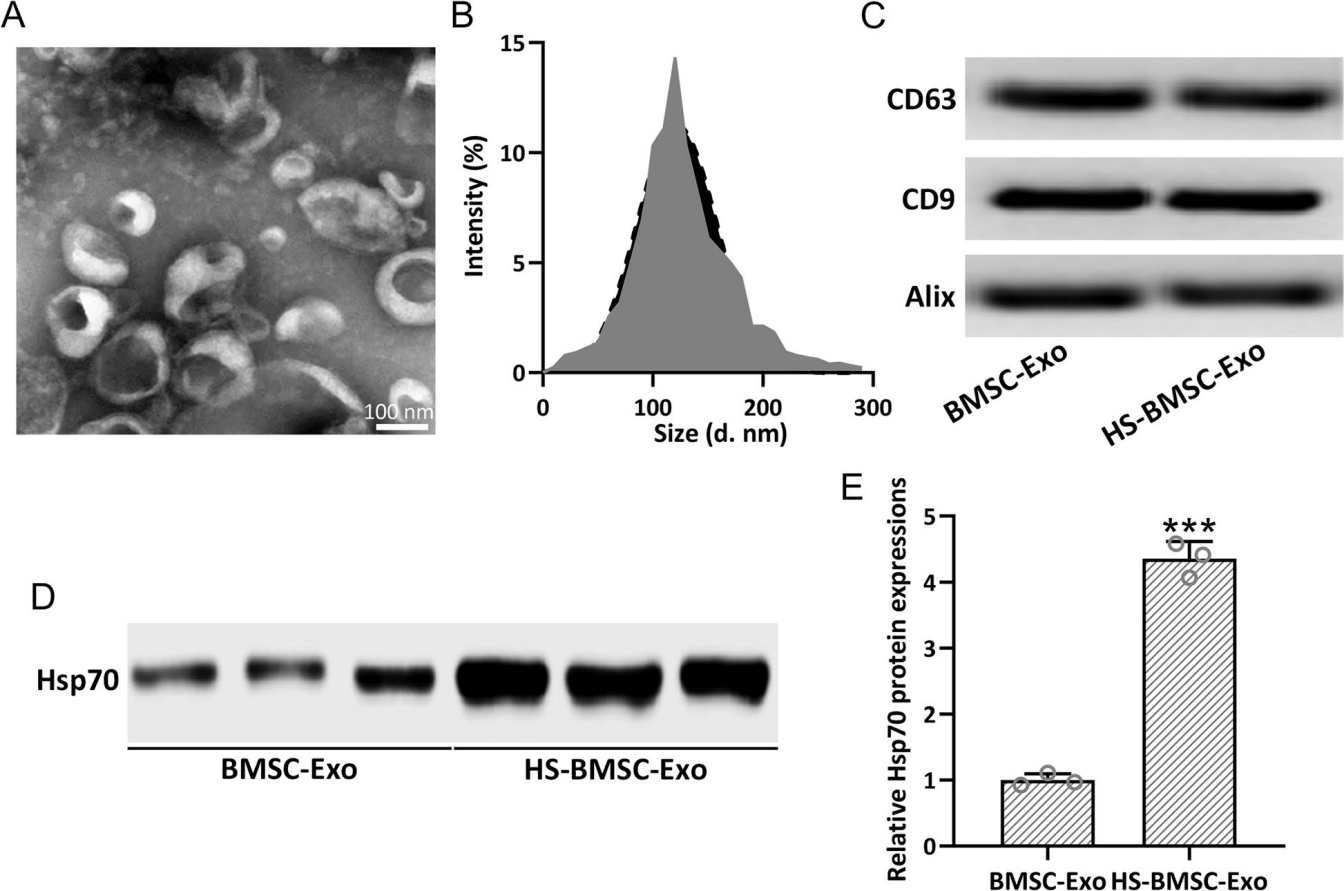
Identification of BMSCs derived exosomes. Transmission electron microscopy (TEM) image of BMSCs derived exosomes (BMSC-Exo) (**A**). Size distribution of BMSC-Exo by nanoparticle tracking analysis (**B**). Detection of CD9, CD63, and Alix by Western blot in BMSC-Exo and heat shock preconditioned BMSCs derived exosomes (HB-BMSC-Exo) (**C**). Detection of HSP70 by Western blot in BMSC-Exo and HB-BMSC-Exo (**D**). The data were shown with mean ± SD. *n* = 3 for each group. ***p < 0.001 compared to BMSC-Exo. Mann Whitney test

To characterize the exosomes derived from heat shock preconditioned BMSCs (HS-BMSC-Exo), the morphology, size, and surface markers were further testified. Single membrane morphology was confirmed by the TEM image (Fig. 2A). The size of BMSCs derived exosomes (BMSC-Exo) was 120 nm, as detected with ZetaView NTA (Fig. 2B). The markers of exosomes, CD63, CD9, and Alix, were detected in both BMSC-Exo and HS-BMSC-Exo (Fig. 2C). As expected, up-regulated HSP70 expression was observed in HS-BMSC-Exo compared with BMSC-Exo (Fig. 2D and E). All of these data demonstrated that heat shock preconditioned BMSCs could produce HSP70 enriched exosomes, which could be utilized to deliver HSP70 to alleviate NLRP3 inflammasomes mediated inflammation.

### HS-BMSC-Exo ameliorate auditory sensitivity in cisplatin exposed mice

In our previous report, we demonstrated that intrathecal infusion of cochlear spiral ganglion progenitor cells derived exosomes (1.2 μg/μL, 1 μL) could significantly reduce cochlea damage in ischemia–reperfusion injury mice [20]. Accordingly, we chose the dose of 1.2 μg/μL for further trans-tympanical injection of exosomes thirty minutes after the induction of cisplatin.

**Fig. 3:**
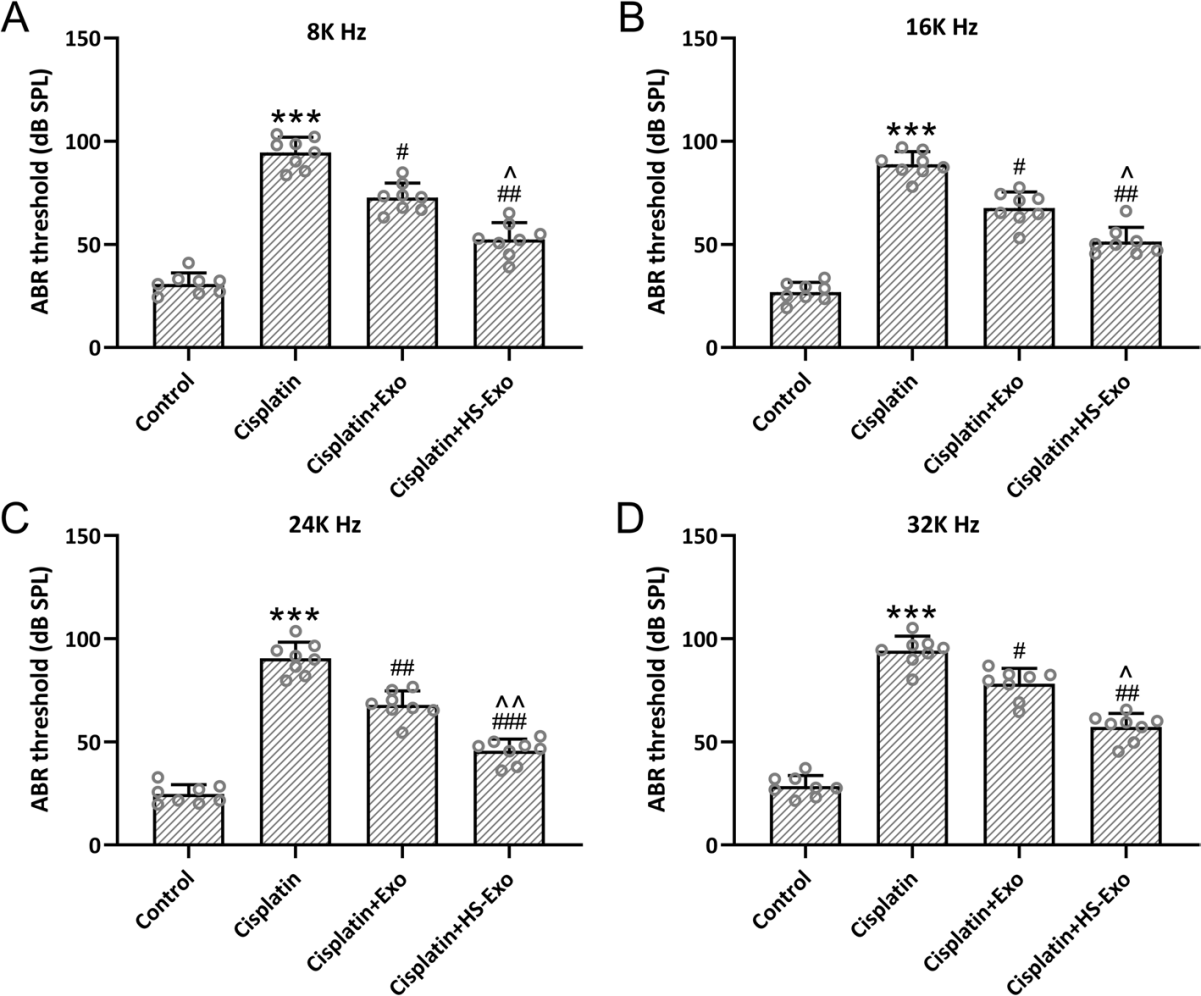
Exosomes from heat shock preconditioned BMSCs ameliorated cisplatin-induced hearing sensitivity in vivo. **A-D** Auditory brainstem response (ABR) revealed that HB-BMSC-Exo reduced the hearing thresholds at 8, 16, 24, and 32 kHz in cisplatin-injected mice. The data were shown with mean ± SD. *n* = 8 for each group. ***p < 0.001 compared to control, #*p* < 0.05, ##*p* < 0.01, ###*p* < 0.001 compared to Cisplatin, ^*p* < 0.05, ^^*p* < 0.01 compared to Cisplatin + Exo. One-way ANOVA followed Tukey’s multiple comparisons test

ABR measurement was utilized to reveal the auditory sensitivity. Hearing thresholds were significantly elevated 7 days after cisplatin exposure at 8 kHz (Fig. 3A), 16 kHz (Fig. 3B), 24 kHz (Fig. 3C), and 32 kHz (Fig. 3D), whereas HS-BMSC-Exo or BMSC-Exo treatment could diminish the thresholds significantly. It was worth noting that HS-BMSC-Exo could dramatically decrease the hearing thresholds at the four frequencies detected compared with BMSC-Exo. All of these indicated that the enriched HSP70 content in the HS-BMSC-Exo might contribute to the increased auditory sensitivity in cisplatin exposed mice.

### HS-BMSC-Exo ameliorate hair cells loss in cisplatin exposed mice

**Fig. 4:**
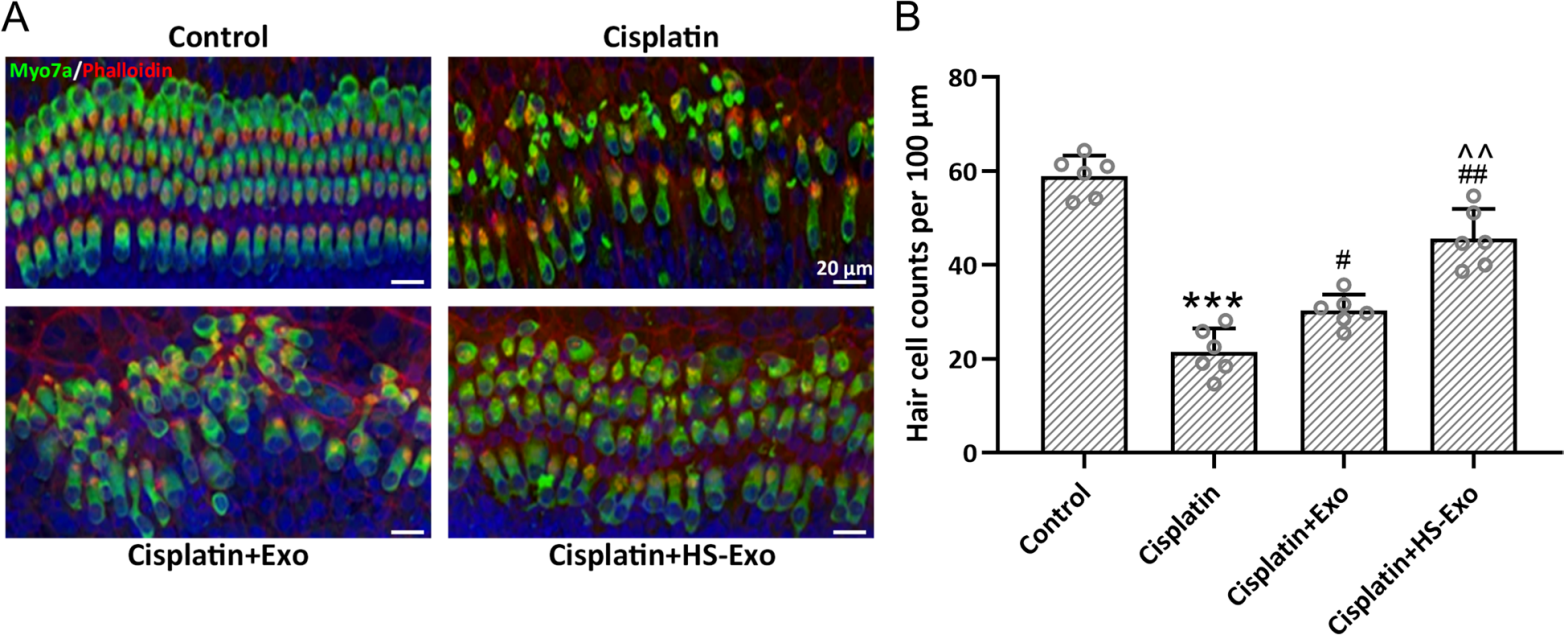
Exosomes from heat shock preconditioned BMSCs ameliorated cisplatin-induced hair cell loss in the middle turns of the cochlea. Representative Myosin 7a staining in different groups (**A**) and quantification of hair cells (**B**). The data were shown with mean ± SD. Eight fields of each mice and six mice in each group were counted. ***p < 0.001 compared to control, #*p* < 0.05, ##*p* < 0.01 compared to Cisplatin, ^^*p* < 0.01 compared to Cisplatin + Exo. One-way ANOVA followed Tukey’s multiple comparisons test

A conspicuous loss of mature hair cells was observed in the middle turn of the cochlea after cisplatin exposure, which could be prevented by the administration of HS-BMSC-Exo or BMSC-Exo (Fig. 4A and B). Compared with BMSC-Exo treatment, HS-BMSC-Exo could further alleviate the loss of hair cells. These results demonstrated that enriched HSP70 content in the HS-BMSC-Exo might contribute to the protection of hair cells loss in cisplatin exposed mice.

### HS-BMSC-Exo alleviate cisplatin-induced inflammation

**Fig. 5:**
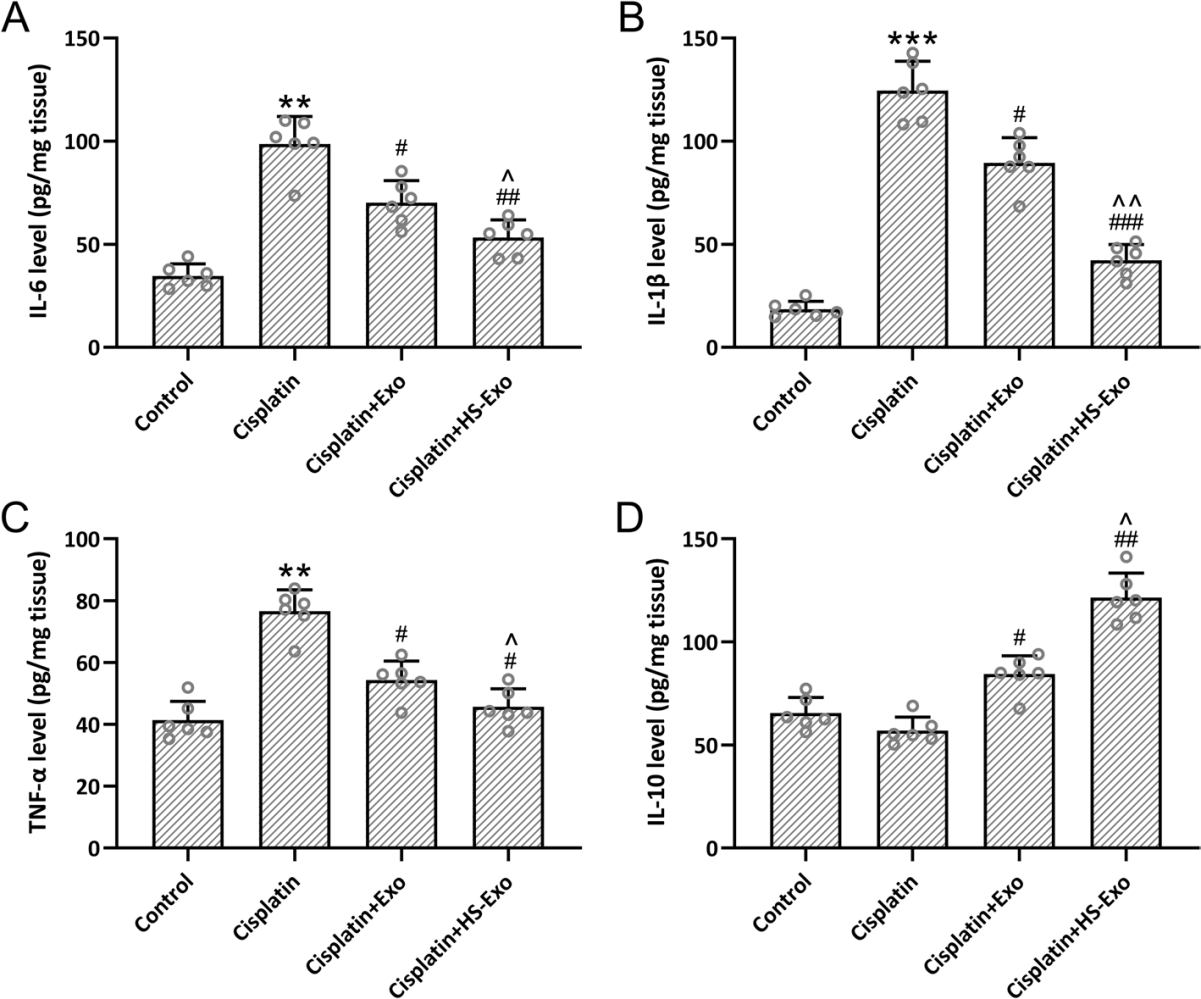
Exosomes from heat shock preconditioned BMSCs ameliorated cisplatin-induced inflammatory responses in the cochlea. ELISA was used to measure the cochlear concentrations of interleukin (IL)-6 (**A**), IL-1β (**B**), tumor necrosis factor (TNF)-α (**C**) and IL-10 (**D**). The data were shown with mean ± SD. *n* = 6 for each group. **p < 0.01, ***p < 0.001 compared to control, #*p* < 0.05, ##*p* < 0.01, ###*p* < 0.001 compared to Cisplatin, ^*p* < 0.05, ^^*p* < 0.01 compared to Cisplatin + Exo. One-way ANOVA followed Tukey’s multiple comparisons test

Cisplatin exposed mice demonstrated up-regulated IL-6 (Fig. 5A), IL-1β (Fig. 5B), TNF-α (Fig. 5C), and down-regulated IL-10 (Fig. 5D), which could be reversed by the administration of HS-BMSC-Exo or BMSC-Exo. Compared with BMSC-Exo treatment, HS-BMSC-Exo could further up-regulate the relative expression of regulatory cytokine of IL-10 and down-regulate the expression of pro-inflammatory cytokines of IL-6, IL-1β, and TNF-α. All of these data indicated that the enriched HSP70 content in HS-BMSC-Exo could favor the anti-inflammation effect.

### HS-BMSC-Exo ameliorate cisplatin-induced cochlear NLRP3 inflammasome activation

**Fig. 6:**
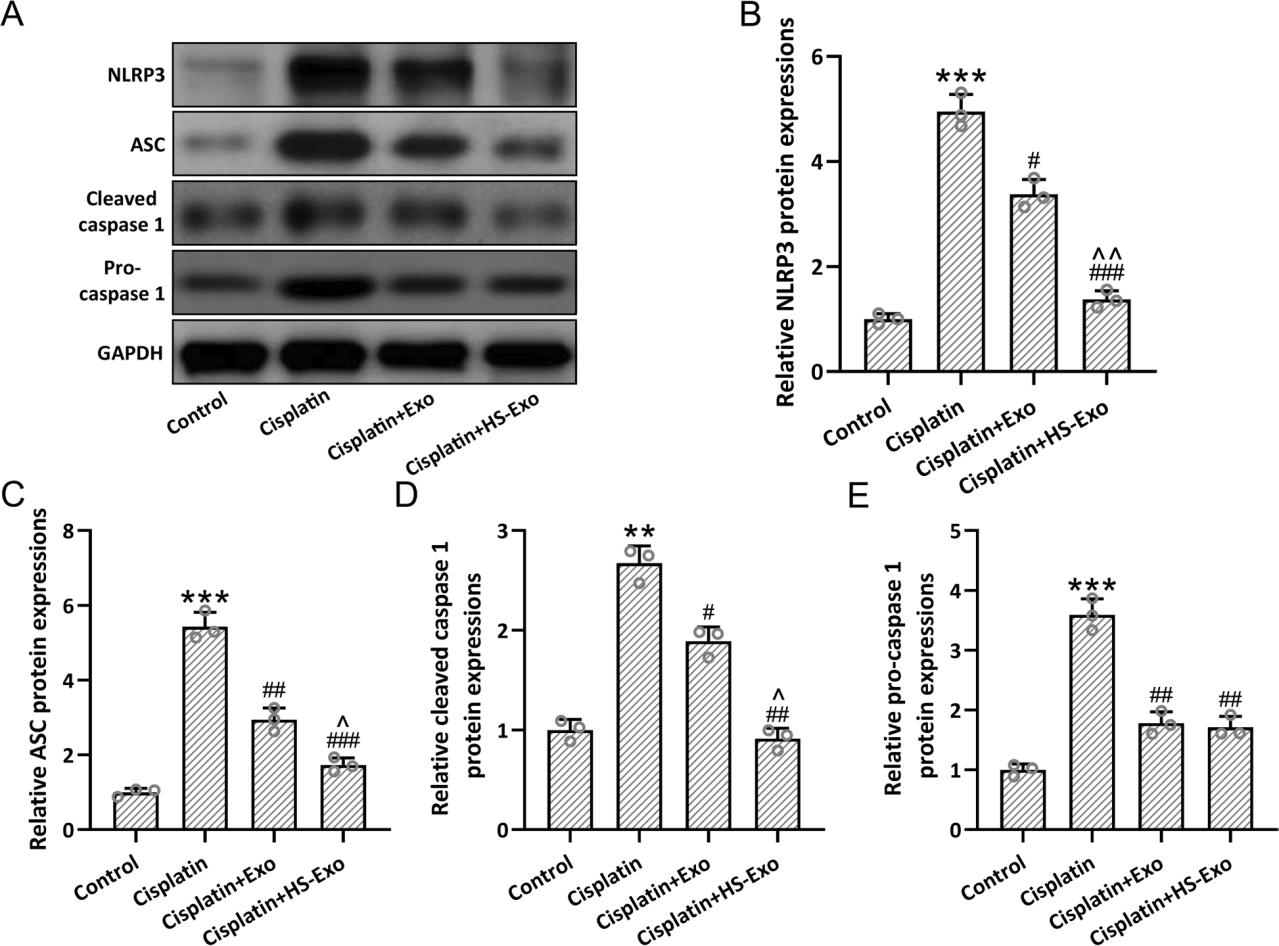
Exosomes from heat shock preconditioned BMSCs ameliorated cisplatin-induced cochlear NLRP3 inflammasome activation. NLRP3, ASC, cleaved caspase-1, and pro-caspase-1 expression in the cochlear tissues were detected with Western blotting (**A**). The expressions were normalized to internal control (**B-E**). The data were shown with mean ± SD. The experiments were repeated 3 times with homogenate from 6 mice in each group. **p < 0.01, ***p < 0.001 compared to control, #*p* < 0.05, ##*p* < 0.01 compared to Cisplatin, ^*p* < 0.05, ^^*p* < 0.01 compared to Cisplatin + Exo. One-way ANOVA followed Tukey’s multiple comparisons test

Western blot was utilized to detect NLRP3 inflammasome activation-related molecules (Fig. 6A). Up-regulated NLRP3 (Fig. 6B), ASC (Fig. 6C), cleaved caspase-1 (Fig. 6D), and pro-caspase-1 (Fig. 6E) could be detected after cisplatin exposure, which could be alleviated by the administration of HS-BMSC-Exo or BMSC-Exo. As expected, the more significant down-regulation could be detected in HS-BMSC-Exo when compared with BMSC-Exo. These results demonstrated that HSP70 enriched exosomes could inhibit the activation of NLRP3 inflammasome.

## Discussion

For the first time, we find that NLRP3 inflammasome activation may contribute to the development of cisplatin-induced inflammatory ototoxicity. Heat shock preconditioned BMSCs could release HSP70 enriched exosomes, which could be utilized to target NLRP3 inflammasome mediated ototoxicity induced by cisplatin with increased auditory sensitivity and alleviated hair cells loss. All of these results demonstrate that heat shock precondition could be utilized to produce HSP70 enriched exosomes to relieve NLRP3 inflammasome mediated inflammation. Our study also indicates the potential treatment strategy to alleviate cisplatin-induced inflammatory ototoxicity when targeting NLRP3 inflammasome activation.

Cisplatin could activate extracellular signal-regulated kinases to prime NLRP3 inflammasomes to induce the release of pro-inflammatory cytokines in various auditory cells [4, 21]. Intraperitoneal administration of cisplatin could activate the NLRP3 inflammasome to cause acute liver and kidney injury [14]. It is further revealed that selective inhibition of NLRP3 inflammasome could attenuate cisplatin-induced renal fibrosis with diminished oxidative stress and inflammation [8]. While, genetic NLRP3 deficiency mice are resistant to ischemic but not cisplatin-induced acute kidney injury and fibrosis (Kim et al., 2013).

In this study, we find that exosomal HSP70 induced by heat shock precondition could alleviate inflammatory cisplatin ototoxicity in mice. As indicated in the previous report, exosomal HSP70 can interact with Toll-like receptor 4 (TLR4) on hair cells to mediate the specific protective effect upon neomycin exposure [11]. All of these results indicate that the interaction between HSP70 and TLR4 should be considered in considering NLRP3 inflammasome target treatment.

Inflammasome activation can recruit and cleave pro-caspase-1 to form cleaved caspase-1, which could subsequently convert pro-IL-1β and pro-IL-18 into mature forms [3, 12, 16]. In this study, decreased IL-1β, IL-6, TNF-α, and increased IL-10 were observed after exosomal HSP70 treatment. NLRP3 inflammasome-driven joint destruction could be regulated by IL-10 in inflammatory arthritis [5]. Acute, lipopolysaccharide exposure induced IL-10 could dampen NLRP3 inflammasome activation in macrophages to avoid overt inflammation [6]. It is worth noting that HSP70-containing extracellular vesicles could induce CD8-positive response and accumulation of anti-tumor cytokine in melanoma and colon carcinoma mice models [7]. The precise exosomal HSP70 targeted cells in cochlear tissues should be investigated in future study.

Some limitations should also be indicated here. The treatment benefit is only observed seven days after the administration of cisplatin, and the chronic treatment effect of HS-BMSC-Exo in cisplatin-induced ototoxicity in mice is not deciphered in this study. There is little control that can be performed to standardize the induction process of exosomal HSP70, and a more dedicated quantifying method to construct HS-BMSC-Exo is performed. Whether HS-BMSC-Exo can be utilized to relieve NLRP3 inflammasome-mediated inflammatory condition is an interesting question worth further deciphering.

## Conclusion

Ototoxicity induced by cisplatin is an obstacle to the effective treatment of tumors. Our study demonstrates that heat shock precondition may provide new potential therapeutic options for BMSCs in regenerative medicine, and HS-BMSC-Exo could be considered as a treatment option in the alleviation of cisplatin-induced ototoxicity.

## Data Availability

Data is available from the authors by request.
